# Kyphoplasty versus vertebroplasty for osteoporotic vertebral compression fractures: a systematic review and meta-analysis of randomized trials

**DOI:** 10.1590/1806-9282.20260411

**Published:** 2026-08-17

**Authors:** Alberto Oliveira, Fabio Veiga de Castro Sparapani, Sérgio Cavalheiro, Franz Jooji Onishi

**Affiliations:** 1 Universidade Federal de São Paulo, Department of Neurosurgery – São Paulo (SP), Brazil.

## INTRODUCTION

Osteoporotic vertebral compression fractures (OVCFs) represent one of the most common and clinically significant complications of osteoporosis, particularly in the elderly population. These fractures are frequently associated with severe axial pain, reduced mobility, progressive spinal deformity, loss of independence, and substantial impairment in quality of life. Beyond pain itself, vertebral collapse may contribute to sagittal imbalance, progressive kyphotic deformity, reduced pulmonary capacity, impaired gastrointestinal function, and increased risk of subsequent fragility fractures. In frail elderly patients, prolonged immobilization secondary to pain may also result in muscle wasting, venous thromboembolism, respiratory complications, and overall functional decline. Consequently, osteoporotic vertebral fractures are associated not only with increased morbidity, but also with higher healthcare utilization, hospitalization rates, and socioeconomic burden worldwide. With the progressive aging of the global population, the incidence of osteoporotic vertebral fractures has increased substantially, making them an important public health concern. Vertebral compression fractures are among the most prevalent osteoporotic fractures and are recognized as a major predictor of future fractures, contributing to a cycle of progressive spinal deformity and functional deterioration. Despite their high prevalence, many vertebral fractures remain underdiagnosed or undertreated, particularly in patients presenting with nonspecific back pain or gradual loss of mobility. Therefore, early diagnosis and appropriate management of symptomatic OVCFs have become increasingly important to improve pain control, preserve mobility, and maintain quality of life^1-3^.

Percutaneous vertebral augmentation techniques have been widely adopted as minimally invasive treatments for pain relief and vertebral stabilization in patients with persistent symptoms despite conservative management. Vertebroplasty consists of percutaneous injection of polymethylmethacrylate cement directly into the vertebral body, providing mechanical stabilization of the fractured vertebra and often resulting in rapid pain improvement. Balloon kyphoplasty was later developed as an evolution of vertebroplasty, aiming to address one of the major concerns associated with vertebral augmentation procedures: cement leakage beyond the vertebral body. In kyphoplasty, an inflatable balloon is inserted before cement injection to create an intravertebral cavity and partially restore vertebral height. This technique allows the use of higher-viscosity cement injected under lower pressure, theoretically reducing the risk of cement extravasation while also potentially improving kyphotic deformity and vertebral body collapse^4-7^.

Cement leakage remains the most frequently reported procedural complication of vertebral augmentation techniques. Although many cases are clinically asymptomatic, cement extravasation may occasionally result in severe complications, including radiculopathy, spinal canal compromise, pulmonary embolism, and vascular injury. From this perspective, kyphoplasty was developed not only as a stabilizing procedure but also as a technical refinement intended to improve procedural safety by reducing uncontrolled cement dissemination outside the vertebral body. Although both vertebroplasty and kyphoplasty are widely used in clinical practice, their comparative efficacy and safety remain debated. Previous observational studies and non-randomized series have reported heterogeneous results regarding pain relief, functional recovery, vertebral height restoration, adjacent fractures, cement leakage, and overall complication rates. Furthermore, many studies are limited by selection bias, heterogeneity in fracture chronicity, differences in patient populations, and variability in procedural techniques^2,6,8,9^.

Therefore, the present systematic review and meta-analysis was restricted to randomized controlled trials in order to provide a more robust and methodologically rigorous comparison between balloon kyphoplasty and vertebroplasty. This study aimed to compare the clinical effectiveness and safety of these vertebral augmentation techniques in adult patients predominantly presenting osteoporotic vertebral compression fractures. Primary outcomes included pain reduction and functional improvement, while secondary outcomes comprised cement leakage, complications, and operative time.

## METHODS

This systematic review and meta-analysis were conducted in accordance with the Preferred Reporting Items for Systematic Reviews and Meta-Analyses (PRISMA) guidelines. The study protocol was prospectively registered in the International Prospective Register of Systematic Reviews (PROSPERO) under registration number CRD420261325378.

This study is a systematic review based exclusively on previously published data and publicly available information. According to the Brazilian National Health Council Resolution No. 510/2016, studies using publicly available data that do not involve direct human participation do not require approval from a Research Ethics Committee.

Randomized controlled trials directly comparing balloon kyphoplasty and vertebroplasty in adult patients (≥18 years) with osteoporotic vertebral compression fractures were eligible for inclusion. Studies were required to report at least one predefined outcome. No restrictions regarding language or publication date were applied. Non-randomized studies, observational cohorts, case series, case reports, biomechanical studies, animal experiments, and review articles were excluded.

A systematic search was performed in PubMed/MEDLINE, Embase, and Google Scholar from database inception to February 26, 2026. Search strategies combined controlled vocabulary and free-text terms related to kyphoplasty, vertebroplasty, osteoporotic fracture, and randomized trials. Mesh and Emtree terms were used for the literature search, using boolean connectors.

Two reviewers independently screened titles and abstracts for potential eligibility, followed by full-text evaluation according to predefined inclusion criteria. Data extraction was performed independently using a standardized form.

The primary outcomes were pain reduction measured by the Visual Analog Scale (VAS), functional outcome assessed by the Oswestry Disability Index (ODI), cement leakage, and overall complication rate. The secondary outcome was operative time.

Risk of bias was assessed using the Cochrane Risk of Bias tool version 2 (RoB 2)^10^. Statistical heterogeneity was evaluated using the I² statistic, and meta-analyses were performed using a random-effects model when significant heterogeneity was detected.

## RESULTS

The EMBASE search yielded 159 records, while the PubMed search retrieved 379 records. In addition to database searches, a complementary search was conducted in Google Scholar to enhance sensitivity and minimize the risk of publication bias. Using the keywords "kyphoplasty," "vertebroplasty," and "randomized," the first 300 studies sorted by relevance were screened for potential eligibility, with an initial sum of 838 studies.

**Figure 1:**
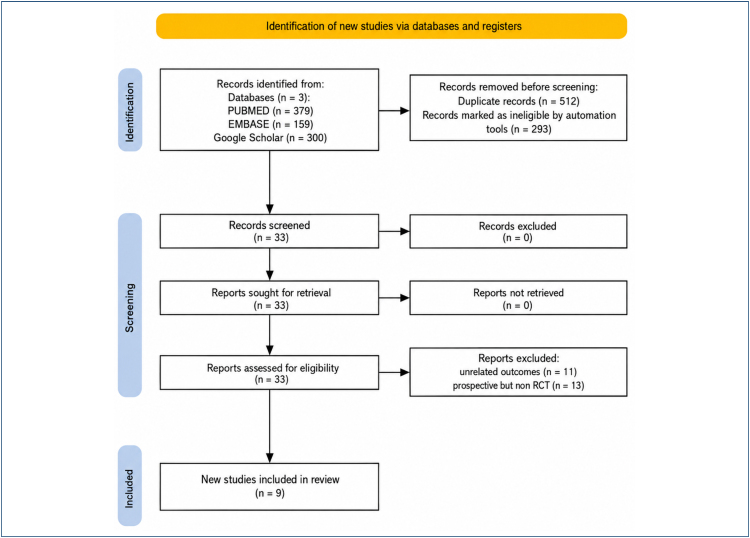
Prisma flow diagram.

A total of nine randomized controlled trials (RCTs)^11-19^ published between 2010 and 2023, were included in this systematic review, encompassing 1,116 patients with osteoporotic vertebral compression fractures treated with either balloon kyphoplasty or vertebroplasty. The included studies originated from diverse geographic regions, reflecting a broad international representation. The trials were conducted in Taiwan, Germany (two studies), the United States (two studies), China (two studies), Italy, and India, encompassing populations from Asia, Europe, and North America (Figure 1).

The mean age of the pooled population was 71.6 years, reflecting the typical epidemiological profile of osteoporotic vertebral compression fractures. Importantly, no relevant differences in baseline age were reported between patients undergoing vertebroplasty and those treated with kyphoplasty across the included studies.

Regarding fracture location, 406 thoracic levels and 505 lumbar levels were treated. In three studies, the exact anatomical distribution of treated levels was not available.

Follow-up duration varied among the included trials. The mean follow-up period was approximately 6 months, although several studies extended clinical assessment to 12 months or longer, allowing evaluation of mid-term outcomes.

Risk of bias for the included studies was assessed using the Cochrane Risk of Bias 2 (RoB 2) tool. Most studies were judged as presenting some concerns, primarily due to insufficient reporting of allocation concealment, lack of blinding inherent to procedural interventions, and limited information regarding pre-specified analysis plans. Three studies (Endres et al.^12^, Dohm et al.^14^, and Griffoni et al.^18^) were classified as high risk of bias, mainly due to issues in the randomization process or substantial missing outcome data. In particular, Endres et al. and Jindal et al. reported prospective allocation but provided insufficient methodological detail regarding true random sequence generation or allocation concealment, resulting in a high-risk judgment in the randomization domain. Overall, the methodological quality of the evidence was considered moderate, with most limitations related to reporting transparency and attrition (Table 1).

**Table 1:** ROB2 risk of bias assessment^10^ showing a moderate to high overall risk of bias of the studies.

Study	Randomization process	Deviations from intended interventions	Missing outcome data	Measurement of outcome	Selection of reported result	Overall risk of bias
Liu 2010	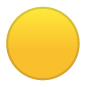	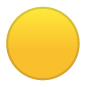	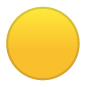	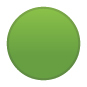	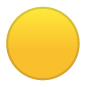	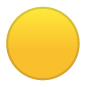
Endres 2012	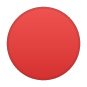	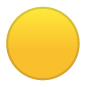	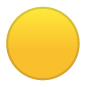	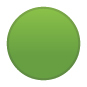	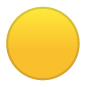	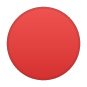
Vogl 2013	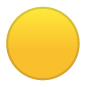	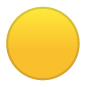	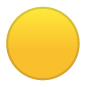	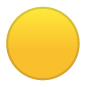	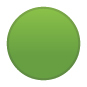	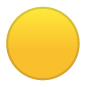
Dohm 2014	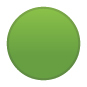	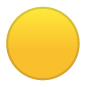	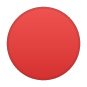	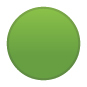	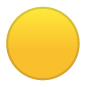	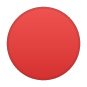
Yi 2014	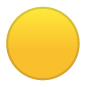	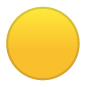	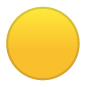	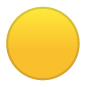	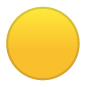	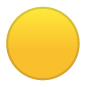
Evans 2015	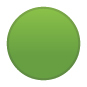	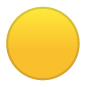	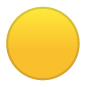	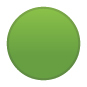	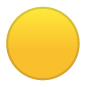	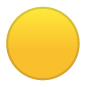
Zhou 2015	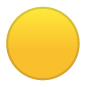	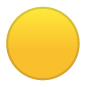	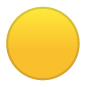	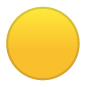	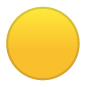	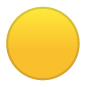
Jindal 2019	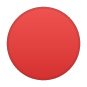	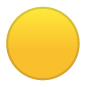	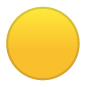	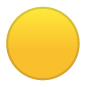	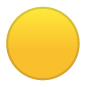	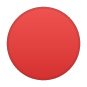
Griffoni 2020	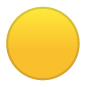	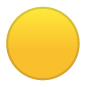	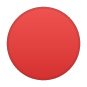	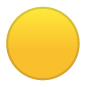	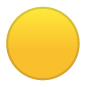	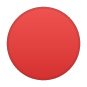

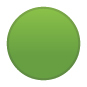
 Low risk; 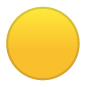 someconcerts; 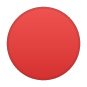 high risk.

### Outcomes analysis

#### Visual Analog Scale

Seven studies reported pain outcomes measured by the VAS. Overall, both balloon kyphoplasty and vertebroplasty demonstrated substantial reductions in pain scores following treatment.

Most trials showed comparable improvements between the two techniques. However, the study by Evans et al. presented a divergent pattern, being the only study suggesting a greater reduction in pain in the vertebroplasty group.

In the pooled analysis, a marginal difference favoring kyphoplasty was observed. Sensitivity analysis was subsequently performed to evaluate the influence of individual studies on the overall effect. After exclusion of the Evans et al. trial, the meta-analysis demonstrated a clearer benefit in favor of kyphoplasty regarding the magnitude of pain reduction measured by VAS.

#### Oswestry Disability Index

Five studies reported functional outcomes using the ODI. Both balloon kyphoplasty and vertebroplasty were associated with improvements in functional status following treatment.

In the pooled analysis, there was a trend toward greater functional improvement in the kyphoplasty group compared with vertebroplasty. However, this difference did not reach statistical significance, suggesting that both procedures provide comparable functional recovery in most patients.

#### Cement leakage

Six studies evaluated the risk of cement leakage. In this outcome, balloon kyphoplasty demonstrated a clear advantage compared with vertebroplasty.

**Figure 2:**
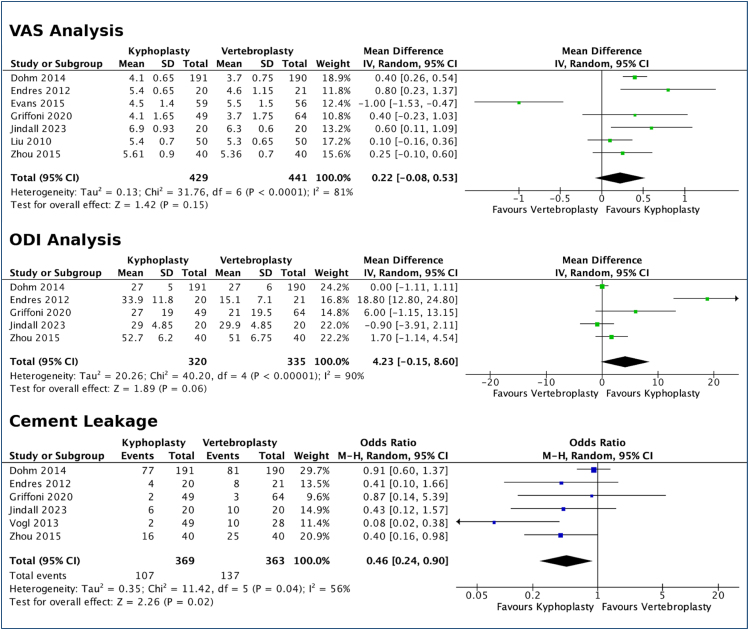
Meta-analysis of Visual Analog Scale, Oswestry Disability Index, and cement leakage. Visual Analog Scale and Oswestry Disability Index showed a non-significant trend favoring kyphoplasty. Cement leakage rates were significantly lower with kyphoplasty compared with vertebroplasty.

Across all included studies, the results were consistent and homogeneous, showing lower rates of cement leakage in the kyphoplasty group. This finding likely reflects the procedural characteristics of kyphoplasty, in which cavity creation allows injection of higher-viscosity cement under lower pressure, thereby reducing the risk of extravasation (Figure 2).

#### General complications

Four studies reported overall clinical complication rates. When analyzed collectively, no statistically significant difference was observed between balloon kyphoplasty and vertebroplasty.

Across the included studies, the incidence of serious complications was low. Notably, only the study by Dohm et al. reported cases of pulmonary embolism, with one event occurring in each treatment group, suggesting no clear difference in embolic risk between the two procedures.

#### Operative time

Operative time was reported in three studies. Overall, balloon kyphoplasty was associated with longer procedural times compared with vertebroplasty, with a mean operative time of 48 min for kyphoplasty versus 37 min for vertebroplasty. This difference likely reflects the additional technical steps required for kyphoplasty, including balloon insertion and cavity creation before cement injection.

## DISCUSSION

Vertebral augmentation procedures have become widely adopted for the management of painful osteoporotic vertebral compression fractures, particularly in patients with persistent symptoms despite conservative therapy. Both vertebroplasty and kyphoplasty aim to stabilize the fractured vertebral body, providing rapid pain relief and facilitating early mobilization in a typically elderly and fragile patient population^9,20,21^.

Kyphoplasty was developed as a refinement of vertebroplasty, primarily to address concerns related to cement leakage, which remains one of the most frequent complications associated with vertebral augmentation procedures. By creating an intravertebral cavity through balloon inflation before cement injection, kyphoplasty enables the administration of more viscous cement under lower injection pressure, thereby reducing the risk of uncontrolled cement extravasation outside the vertebral body. Although many cement leaks are clinically asymptomatic, cement extravasation may occasionally result in severe complications, including radiculopathy, spinal canal compromise, pulmonary embolism, and vascular injury. From a safety perspective, the findings of the present meta-analysis reinforce the concept that kyphoplasty demonstrates a more favorable safety profile regarding cement leakage when compared with vertebroplasty, with consistently lower leakage rates across the included randomized studies^9,13,22^.

Another frequently debated outcome is the occurrence of adjacent vertebral fractures. Although biomechanical models suggest that cement augmentation may alter load transmission within the spinal column, clinical evidence remains inconclusive. Osteoporosis itself carries a high intrinsic risk of subsequent fractures, making it difficult to establish a direct causal relationship between vertebral augmentation procedures and adjacent-level fractures^23,24^. Similarly, several studies have focused on radiographic outcomes such as vertebral height restoration and kyphotic angle correction, which are generally more pronounced after kyphoplasty. However, the clinical significance of these radiological improvements remains uncertain, as better vertebral alignment does not consistently correlate with superior long-term functional recovery or patient-reported outcomes^25-27^.

In this context, clinical outcomes—particularly pain relief and functional improvement—remain the most relevant endpoints in this fragile population. The findings of the present meta-analysis suggest that both vertebroplasty and kyphoplasty provide substantial clinical improvement in patients with osteoporotic vertebral compression fractures. Nevertheless, kyphoplasty demonstrated a tendency toward superior clinical outcomes and, more importantly, significantly lower cement leakage rates, supporting its role as a safe and effective vertebral augmentation technique with a potentially more favorable procedural safety profile.

### Study limitations

This study has some limitations that should be acknowledged. First, the number of randomized controlled trials directly comparing balloon kyphoplasty and vertebroplasty remains limited, restricting the overall sample size and statistical power of the pooled analyses. Second, there was heterogeneity among the included studies regarding the timing of intervention, as some patients were treated in the acute phase whereas others underwent vertebral augmentation later in the disease course. This is relevant because current evidence suggests that earlier intervention, particularly within the first 4 weeks after fracture onset, may be associated with greater pain relief and kyphotic angle correction. In addition, several studies presented methodological concerns, including incomplete reporting of randomization procedures, lack of blinding inherent to procedural interventions, and losses during follow-up. Finally, the low incidence of reported complications may have limited the ability to detect small differences in safety outcomes between the two procedures.

## CONCLUSION

This systematic review and meta-analysis indicate that balloon kyphoplasty and vertebroplasty provide comparable improvements in pain relief (VAS) and functional outcomes (ODI) in patients with osteoporotic vertebral compression fractures. However, kyphoplasty demonstrated significantly lower rates of cement leakage across the included studies. Given that cement leakage may lead to clinically relevant complications, this finding represents an important safety advantage. Although kyphoplasty was associated with slightly longer operative times, this difference appears modest and unlikely to be clinically meaningful. Overall, the available evidence supports kyphoplasty as a safe and effective therapeutic option, offering similar clinical efficacy with a more favorable safety profile compared with vertebroplasty.

## Data Availability

The datasets generated and/or analyzed during the current study are available from the corresponding author upon reasonable request.
